# Interprofessional Collaboration and Care for Older Adults through the 5M’s: Workshop Development/Implementation

**DOI:** 10.1093/geroni/igaf122.4301

**Published:** 2025-12-31

**Authors:** Philip Solomon, Marie Dobrayel, Michele Pisano, Kaitlin Tangredi, Barbara Vogel, Christian Nouryan, Edith Burns

**Affiliations:** Northwell Health, New Hyde Park, New York, United States; Northwell Health, New Hyde Park, New York, New York, United States; St. John’s University, Queens, New York, United States; Northwell Health, New Hyde Park, New York, New York, United States; Northwell Health, New Hyde Park, New York, New York, United States; Northwell Health, Great Neck, New York, United States; Zucker School of Medicine Hofstra-Northwell, Manhasset, New York, United States

## Abstract

The field of Geriatrics is inherently based on interprofessional collaboration, requiring a variety of health professionals to address the principles and complexity of caring for older adults. A novel workshop curriculum was developed and implemented across a large health system weaving three threads: the 5M’s framework, the Interprofessional Education Collaborative (IPEC) core competencies, and health system navigation for patients and caregivers. Stakeholders in numerous health professions were included in content creation and learner recruitment. The interactive, half-day, in-person sessions reframed each “M” in the context of health system navigation: 1) Medications: How do older adults pay for/access them? 2) Mobility: What is the proper venue to care for older adults with debility? 3) Mind: How do we provide support and coverage for custodial care for older adults with dementia? 4) What Matters Most: How can a health system align care and support patients’ goals? 5) Multicomplexity: How can we work collaboratively to care for older adults and help them navigate complex health systems? This paper reports results of > 200 learners from nine health professions who have taken the course. Curriculum evaluation was guided by the Kirkpatrick model, assessing learners’ attitudes, knowledge, and behavior. Over 93% felt knowledge would help both clinical practice and professional development. There were statistically significant increases in knowledge of IPEC competencies, ability to define roles of other health professions, intent to apply 5M’s in clinical practice, and interest in pursuing formal Geriatrics training. Next steps include further internal/external dissemination and adaptation (e.g., asynchronous learning modules).

